# Association of Neuropeptide Y (*NPY*), Interleukin-1B (*IL1B*) Genetic Variants and Correlation of *IL1B* Transcript Levels with Vitiligo Susceptibility

**DOI:** 10.1371/journal.pone.0107020

**Published:** 2014-09-15

**Authors:** Naresh C. Laddha, Mitesh Dwivedi, Mohmmad Shoab Mansuri, Mala Singh, Hetanshi H. Patel, Nishtha Agarwal, Anish M. Shah, Rasheedunnisa Begum

**Affiliations:** Department of Biochemistry, Faculty of Science, The Maharaja Sayajirao University of Baroda, Vadodara, Gujarat, India; University of Tennessee, United States of America

## Abstract

**Background:**

Vitiligo is a depigmenting disorder resulting from loss of functional melanocytes in the skin. NPY plays an important role in induction of immune response by acting on a variety of immune cells. NPY synthesis and release is governed by IL1B. Moreover, genetic variability in *IL1B* is reported to be associated with elevated NPY levels.

**Objectives:**

Aim of the present study was to explore *NPY* promoter −399T/C (rs16147) and exon2 +1128T/C (rs16139) polymorphisms as well as *IL1B* promoter −511C/T (rs16944) polymorphism and to correlate *IL1B* transcript levels with vitiligo.

**Methods:**

PCR-RFLP method was used to genotype *NPY* -399T/C SNP in 454 patients and 1226 controls; +1128T/C SNP in 575 patients and 1279 controls and *IL1B* −511C/T SNP in 448 patients and 785 controls from Gujarat. *IL1B* transcript levels in blood were also assessed in 105 controls and 95 patients using real-time PCR.

**Results:**

Genotype and allele frequencies for *NPY* −399T/C, +1128T/C and *IL1B* −511C/T SNPs differed significantly (*p*<0.0001, *p*<0.0001; *p* = 0.0161, *p* = 0.0035 and *p*<0.0001, *p*<0.0001) between patients and controls. ‘TC’ haplotype containing minor alleles of *NPY* polymorphisms was significantly higher in patients and increased the risk of vitiligo by 2.3 fold (*p*<0.0001). Transcript levels of *IL1B* were significantly higher, in patients compared to controls (*p* = 0.0029), in patients with active than stable vitiligo (*p* = 0.015), also in female patients than male patients (*p* = 0.026). Genotype-phenotype correlation showed moderate association of *IL1B* -511C/T polymorphism with higher *IL1B* transcript levels. Trend analysis revealed significant difference between patients and controls for *IL1B* transcript levels with respect to different genotypes.

**Conclusion:**

Our results suggest that *NPY* −399T/C, +1128T/C and *IL1B* −511C/T polymorphisms are associated with vitiligo and *IL1B* −511C/T SNP influences its transcript levels leading to increased risk for vitiligo in Gujarat population. Up-regulation of *IL1B* transcript in patients advocates its possible role in autoimmune pathogenesis of vitiligo.

## Introduction

Vitiligo is an acquired hypomelanotic disorder characterized by circumscribed depigmented macules in the skin resulting from the loss of functional melanocytes from the cutaneous epidermis. It is frequently associated with positive family history [1]–[2]. The most accepted view in vitiligo pathogenesis is the interaction between genetic and non-genetic factors that influence melanocyte survival and function [3]–[4]. Neurochemical mediators secreted by the nerve endings such as acetylcholine and catecholamines might lead to the destruction of melanocytes [4], [5].

Neuropeptide-Y (NPY) is a 36-amino acid peptide which is able to induce melanocyte dendricity and participates in the regulation of cell substrate adhesion, cell motility and shape [6]. Out of five receptors of NPY, Y1 receptor is present on immune cells [7]. Interleukin-1B (IL1B) is a key regulator of the body's inflammatory responses which exerts numerous biological effects and its defects result in various diseases [8]. IL1B induces biosynthesis and release of NPY which in turn induces the catecholamine release [9]. In order to understand the possible mechanism of action of NPY in vitiligo pathogenesis, neuro-immune-cutaneous system may be considered. NPY is one of the most abundant neuropeptides in the central and peripheral nervous system. Under physiological conditions, NPY is secreted either by sympathetic postganglionic nerve fibers or by activated macrophages and binds to Y1 receptors expressed on B cells, T cells, dendritic cells and macrophages. Interestingly, the NPY system has emerged to have an important role in the induction of a number of immune responses by acting on a variety of immune cells [10]. The possible roles of NPY include immune cell distribution, production of cytokines by T helper cells and release of inflammatory mediators from macrophages [11].

Abnormal release of catecholamines from autonomic nerve endings may play an etiological role in the onset and development of vitiligo by an overproduction of toxic radicals in the microenvironment of melanocytes [5]. Sympathetically-derived NPY is preferentially released, which functions to homeostatically regulate norepinephrine release. Moreover, altered balance of neuropeptides in vitiliginous skin supports the role of nervous system in the pathogenesis of vitiligo [12]. The levels of NPY in the tissue fluids from skin lesions were significantly higher than those from unaffected skin in segmental vitiligo [13]. Thus, NPY might play a critical role in the pathogenesis of vitiligo, *via* neuro-immune mechanism on the melanocytes. Furthermore, immunological and inflammatory processes, endogenous mediators, and cell and tissue injury can trigger interleukin-1 (IL1) production [14]. IL1B is the predominant form of IL1 being produced and secreted by activated macrophages, monocytes, dendritic cells, natural killer (NK) cells and B cells [15], [16], thereby potentially contributing to the autoimmune pathogenesis of vitiligo *via* inflammosome formation.

The human *NPY* gene has two well known single nucleotide polymorphisms (SNPs) i.e., in promoter region, −399 T/C (rs16147) and a non-synonymous polymorphism in exon 2, +1128 T/C (rs16139). Sommer *et al.* [17] suggested that promoter region variation at −399 T/C results in elevated expression of *NPY*. The 1498 bp long *IL1B* gene consists of seven exons and six introns, which encodes for a 269 amino acid protein. IL1B also plays an important role during inflammation, acute phase reaction and apoptosis. *IL1B* −511C/T (rs16944) polymorphism has been reported to be involved in several autoimmune diseases [18].

The present study was designed to explore the role of *NPY* in vitiligo pathogenesis by addressing its non-synonymous (+1128 T/C) and promoter (−399 T/C) polymorphisms. In addition, the study investigated *IL1B* −511C/T promoter polymorphism and its transcript level changes in a case-control approach.

## Materials and Methods

### Study subjects

The study group included 575 vitiligo patients (256 males and 319 females) who referred to S.S.G. Hospital, Vadodara and Civil Hospital, Ahmedabad, Gujarat, India. A total of 1279 unaffected individuals (552 males and 727 females) were included in this study (Table S1). None of the healthy individuals had any evidence of vitiligo and any other disease. The importance of the study was explained to all participants and written consent was obtained from all patients and controls before performing the studies. The study plan and consent forms were approved by the Institutional ethical committee for human research (IECHR), Faculty of Science, The Maharaja Sayajirao University of Baroda, Vadodara, Gujarat, India.

The diagnosis of vitiligo by dermatologists was clinically based on characteristic skin depigmentation with typical localization and white colour on the skin lesions under Woods lamp and confirmed as two different types: generalized vitiligo (including acrofacial vitiligo and vitiligo universalis) (GV) and localized vitiligo (LV). The patients with active vitiligo had existing lesions spreading and/or new lesions had appeared within the previous 6 months, whereas patients with no increase in lesion size or number in last six months were considered as stable vitiligo.

### Genotyping of *NPY* structural and promoter polymorphisms

Genomic DNA was extracted from whole blood using Qiagen DNA extraction kit (Qiagen, USA). PCR-RFLP method was used to genotype +1128 T/C and −399 T/C polymorphisms of *NPY* gene. Partial gene sequence containing *NPY* +1128 T/C SNP was amplified using the forward 5′-ATTGGGGGTCGCGTGTGGTAG-3′ and reverse 5′-GTCCTGCCCTGGGATAGA GCG-3′ primers. Partial gene sequence containing -399 T/C SNP was amplified using the forward 5′-TTCCTACTCCGGCACCCAGTGAG-3′ and reverse 5′-GGGCTTTTATGGAGC TTCCTCGC-3′ primers. The amplified products for +1128 T/C and −399 T/C polymorphisms were 418 bp and 402 bp amplicons respectively. Restriction enzymes *BseN*I and *Alu*I (Fermentas, Vilnius, Lithuania) were used for detection of *NPY* +1128 T/C and −399 T/C SNPs respectively (Figs. S1A & B).

### Genotyping of *IL1B* promoter polymorphism

The *IL1B* −511C/T SNP was genotyped using forward 5′-GTTTAGGAATCTTCCCACTT-3′ and reverse 5′-TGGCATTGATCTGGTTCATC-3′ primers which yielded a 305 bp amplicon. Restriction enzyme *Bsu36*I (New England Biolabs Inc., UK) was used and it cuts the *IL1B* amplicon at the ancestral allele ‘T’ to give 113 bp and 192 bp products but the polymorphic C allele remains uncut (Fig. S1C).

### Determination of *IL1B* and *GAPDH* mRNA expression

#### RNA extraction and cDNA synthesis

Total RNA from whole blood was isolated using Ribopure blood Kit (Ambion inc. Texas, USA) by following the manufacturer's protocol. RNA integrity was verified by 1.5% agarose gel electrophoresis, RNA purity and yield was determined spectrophotometrically at 260/280 nm. RNA was treated with DNase I (Ambion inc. Texas, USA) before cDNA synthesis to avoid DNA contamination. One microgram of total RNA was used to prepare cDNA using the RevertAid First Strand cDNA Synthesis Kit (Fermentas, Vilnius, Lithuania) according to the manufacturer's instructions in the MJ Research Thermal Cycler (Model PTC-200, Watertown, MA, USA).

#### Real-time PCR

The expression of *IL1B* and *GAPDH* transcripts were measured by real-time PCR using SYBR Green method and gene specific forward 5′-AGATGAAGTGCTCCTTCCAGG-3′ and reverse 5′-TGGTCGGAGATTCGTAGCTG-3′ primers (Eurofins, Bangalore, India). *GAPDH* was used as a reference gene and its expression was monitored using forward 5′-ATCCCATCACC ATCTTCCAGGA-3′ and reverse 5′-CAAATGAGCCCCAGCCTTCT-3′ primers (Table S2). Real-time PCR was performed in duplicates in 20 µl volume using LightCycler480 SYBR Green I Master (Roche Diagnostics GmbH, Mannheim, Germany) following the manufacturer's instructions and carried out in the LightCycler480 Real-Time PCR (Roche Diagnostics GmbH, Mannheim, Germany). The thermal cycling conditions included an initial activation step at 95°C for 10 min, followed by 45 cycles of denaturation, annealing and amplification (95°C for 10 sec., 65°C for 20 sec., 72°C for 20 sec.). The fluorescence data collection was performed during the extension step. At the end of the amplification phase a melt curve analysis was carried out to check the specificity of the products formed. The PCR cycle at which PCR amplification begins its exponential phase and product fluorescence intensity finally rises above background and becomes visible was considered as the crossing point (C_P_) or cycle threshold (C_T_). The ΔC_T_ or ΔC_P_ value was determined as the difference between the cycle threshold of target gene (*IL1B*) and reference gene (*GAPDH*). The difference between the two ΔC_P_ values (ΔC_P_ Controls and ΔC_P_ patients) was considered as ΔΔC_P_ to obtain the value of fold expression (2^−ΔΔCp^).

### Statistical analyses

The distribution of the genotypes and allele frequencies of *NPY* and *IL1B* polymorphisms for patients and control subjects were compared using chi-squared test with 3×2 and 2×2 contingency tables respectively using Prism 4 software (Graphpad software Inc; San Diego CA, USA, 2003). *p*-values less than 0.017 were considered as statistically significant due to Bonferroni's correction for multiple testing. Odds ratio (OR) with respective confidence interval (95% CI) for disease susceptibility was also calculated. Haplotype analysis was carried out using http://analysis.bio-x.cn/myAnalysis.php [19]. The linkage disequilibrium (LD) coefficients D′  =  D/Dmax and r^2^-values for the pair of the most common alleles at each site were estimated using the Haploview programe version 4.1 [20]. Age of onset analysis and relative expression of *IL1B* in patient and control groups were plotted and analyzed by Mann-Whitney Wilcoxon test using Prism 4 software (Graphpad software Inc; San Diego CA, USA, 2003). Cochran-Armitage trend test was performed using SAS 9.2 software for analyzing the trend of *IL1B* transcript levels with respect to the genotype for each group individually. Further, ANOVA's trend test was used to compare the mean ΔCp values across the different genotype categories using SPSS version 20 software.

## Results

### Analysis of association between *NPY* exon 2 (+1128 T/C) and promoter (−399 T/C) polymorphisms and vitiligo

We investigated 575 patients and 1279 controls for *NPY* exon 2 (+1128 T/C) polymorphism and 454 patients and 1226 controls for *NPY* promoter (−399 T/C) polymorphism. The genotype and allele frequencies for exon 2 (+1128 T/C) and −399 T/C promoter polymorphisms differed significantly between patients and controls (*p*<0.0001, *p* = 0.0161 and p<0.0001, *p* = 0.0035 respectively) (Table 1). The patient population deviated from Hardy-Weinberg equilibrium (HWE), whereas the control population was found to be in HWE for +1128 T/C (*p*<0.0001 and *p* = 0.7747 respectively) and for −399 T/C polymorphisms (*p*<0.0001 and *p* = 0.0759 respectively) (Table 2). *NPY* exon 2 (+1128; T/C) and promoter (−399; T/C) polymorphisms when compared between generalized (GV) and localized (LV) vitiligo patients (Table 2), the observed genotype frequencies for these polymorphisms in GV and LV patients with respect to unaffected controls were found to be significantly different (*p*<0.0001, *p* = 0.0002 and *p* = 0.029, *p*<0.0001). The allelic frequencies also differed significantly for exon 2 (+1128; T/C) (*p*<0.0001; *p* = 0.029) and promoter (−399; T/C) polymorphisms (*p* = 0.029, *p* = 0.019) respectively for *NPY* gene in GV and LV patients as compared to controls (Table 2). The observed genotype frequencies for the two polymorphisms also differed in active (AV) and stable vitiligo (SV) patients with respect to unaffected controls (*p* = 0.0002, *p*<0.0001 and *p*<0.0001, *p* = 0.0002 respectively) (Table 3). The allelic frequencies for both the above polymorphisms differed significantly in the case of AV patients (*p* = 0.0007 and *p*<0.0001 respectively) compared to unaffected controls. The allelic frequencies of *NPY* promoter polymorphism also differed significantly in SV patients as compared to controls (*p* = 0.0073), but the allelic frequencies of *NPY* exon 2 (+1128; T/C) polymorphism did not differ in stable vitiligo patients as compared to controls (*p* = 0.075).

**Table 1:** Association studies for *NPY* gene exon 2 (+1128 T/C), promoter (−399 T/C) and *IL1B* gene promoter (−511 C/T) polymorphisms in Gujarat vitiligo patients and unaffected controls.

SNP	Genotype or allele	Patients (freq.)	Control (Freq.)	*p* for Association	*p* for HWE	Odds ratio (95% CI)
	**Genotype**	**(n = 575)**	**(n = 1279)**			
**rs16139** *NPY* Exon 2 (+1128 T/C)	TT	495 (0.86)	1130 (0.88)	<0.0001^a^	0.7747 (C) <0.0001 (P)	0.7147 (0.5473–0.9333)
	TC	66 (0.11)	145 (0.11)			
	CC	14 (0.03)	4 (0.01)			
	Allele					
	T	1056 (0.92)	2405 (0.94)	0.0161^b^		
	C	94 (0.08)	153 (0.06)			
	**Genotype**	**(n = 454)**	**(n = 1226)**			
**rs16147** *NPY* Promoter (−399 T/C)	TT	129 (0.28)	347 (0.28)	<0.0001^a^	0.0759 (C) <0.0001 (P)	0.7945 (0.6820–0.9255)
	TC	162 (0.36)	581 (0.47)			
	CC	163 (0.36)	298 (0.24)			
	Allele					
	T	420 (0.46)	1275 (0.52)	0.0035^b^		
	C	488 (0.54)	1177 (0.48)			
	**Genotype**	**(n = 448)**	**(n = 785)**			
**rs16944** *IL1B* Promoter (−511 C/T)	CC	234 (0.52)	489 (0.62)	<0.0001^a^	0.1243(C) 0.0002 (P)	0.5919 (0.4905–0.7143)
	CT	156 (0.35)	270 (0.34)			
	TT	58 (0.13)	26 (0.03)			
	Allele					
	C	624 (0.70)	1248 (0.79)	<0.0001^b^		
	T	272 (0.30)	322 (0.21)			

‘n’ represents number of Patients/ Controls, HWE refers to Hardy-Weinberg Equilibrium, (P) refers to vitiligo patients, (C) refers to healthy control individuals and CI represents Confidence Interval.

a Vitiligo patients vs. Controls using the chi-squared test with 3×2 contingency table,

b Vitiligo patients vs. Controls using the chi-squared test with 2×2 contingency table.

Odds ratio is based on allele frequency distribution. Statistical significance was considered at *p* value ≤0.017 due to Bonferroni's correction for multiple testing.

**Table 2:** Association studies for *NPY* gene exon 2 (+1128 T/C), promoter (−399 T/C) and *IL1B* gene promoter (−511 C/T) polymorphisms in generalized and localized vitiligo patients from Gujarat.

SNP	Genotype or allele	Generalized Vitiligo Patients (Freq.)	Localized Vitiligo Patients (Freq.)	Controls (Freq.)	*p* for Association	*p* for HWE	Odds ratio (95% CI)
	**Genotype**	**(n = 335)**	**(n = 240)**	**(n = 1279)**			
**rs16139** *NPY* Exon 2 (+1128 T/C)	TT	270 (0.81)	225 (0.94)	1130 (0.88)	<0.0001^a^ 0.0002^b^	0.001 (GV) <0.0001 (LV) 0.7747 (C)	0.5074 (0.3774–0.6749) (GV) 0.6479 (0.3980–1.055) (LV)
	TC	55 (0.16)	11 (0.04)	145 (0.11)			
	CC	10 (0.03)	4 (0.02)	4 (0.01)			
	Allele						
	T	595 (0.89)	461 (0.96)	2405 (0.94)	<0.0001^c^		
	C	75 (0.11)	19 (0.04)	153 (0.06)	0.078^d^		
	**Genotype**	**(n = 264)**	**(n = 190)**	**(n = 1226)**			
**rs16147** *NPY* Promoter (−399 T/C)	TT	68 (0.26)	61 (0.32)	347 (0.28)	0.029^a^ <0.0001^b^	0.012 (GV) <0.0001 (LV) 0.0759 (C)	0.8114 (0.6721–0.9797) (GV) 0.7715 (0.6210–0.9584) (LV)
	TC	111 (0.42)	51 (0.27)	581 (0.47)			
	CC	85 (0.32)	78 (0.41)	298 (0.24)			
	Allele						
	T	247 (0.47)	173 (0.46)	1275 (0.52)	0.029^c^		
	C	281 (0.53)	207 (0.54)	1177 (0.48)	0.019^d^		
	**Genotype**	**(n = 261)**	**(n = 187)**	**(n = 785)**			
**rs16944** *IL1B* Promoter (−511 C/T)	CC	125 (0.48)	109 (0.58)	489 (0.62)	<0.0001^a^ 0.0003^b^	0.007 (GV) 0.0145 (LV) 0.1243 (C)	0.5116 (0.4109–0.6370) (GV) 0.7368 (0.5671–0.9573) (LV)
	CT	97 (0.37)	59 (0.32)	270 (0.34)			
	TT	39 (0.15)	19 (0.10)	26 (0.03)			
	Allele						
	C	347 (0.66)	277 (0.74)	1248 (0.79)	<0.0001^c^		
	T	175 (0.34)	97 (0.26)	322 (0.21)	0.0218^d^		

‘n’ represents number of Patients/ Controls, HWE refers to Hardy-Weinberg Equilibrium, (GV) refers to Generalized Vitiligo, (LV) refers to Localized Vitiligo, (C) refers to Controls and CI refers to Confidence Interval.

a Generalized Vitiligo vs. Controls using the chi-squared test with 3×2 contingency table,

b Localized Vitiligo vs. Controls using the chi-squared test with 3×2 contingency table,

c Generalized Vitiligo vs. Controls using the chi-squared test with 2×2 contingency table,

d Localized Vitiligo vs. Controls using the chi-squared test with 2×2 contingency table.

Odds ratio is based on allele frequency distribution. Statistical significance was considered at *p* value ≤0.017 due to Bonferroni's correction for multiple testing.

**Table 3:** Association studies for *NPY* gene exon 2 (+1128 T/C), promoter (−399 T/C) and *IL1B* gene promoter (−511 C/T) polymorphisms in active and stable vitiligo patients from Gujarat.

SNP	Genotype or allele	Active Vitiligo Patients (Freq.)	Stable Vitiligo Patients (Freq.)	Controls (Freq.)	*p* for Association	*p* for HWE	Odds ratio (95% CI)
	**Genotype**	**(n = 756)**	**(n = 394)**	**(n = 1279)**			
**rs16139** *NPY* Exon 2 (+1128 T/C)	TT	314 (0.83)	181(0.94)	1130 (0.88)	0.0002^a^ <0.0001^b^	0.0064 (AV) <0.0001 (SV) 0.7747 (C)	0.6044 (0.451–0.8099) (AV) 1.0757 (0.679–1.7041) (SV)
	TC	56 (0.15)	10 (0.04)	145 (0.11)			
	CC	8 (0.02)	6 (0.02)	4 (0.01)			
	Allele						
	T	684 (0.90)	372 (0.94)	2405 (0.94)	0.0007^c^		
	C	72 (0.10)	22 (0.06)	153 (0.06)	0.075^d^		
	**Genotype**	**(n = 285)**	**(n = 169)**	**(n = 1226)**			
**rs16147** *NPY* Promoter (−399 T/C)	TT	56 (0.20)	73 (0.43)	347 (0.28)	<0.0001^a^ 0.0002^b^	0.0003 (AV) 0.0001 (SV) 0.0759 (C)	0.5717 (0.4744–0.689) (AV) 1.3711 (1.0879–1.7281) (SV)
	TC	106 (0.37)	56 (0.33)	581 (0.47)			
	CC	123 (0.43)	40 (0.24)	298 (0.24)			
	Allele						
	T	218 (0.38)	202 (0.59)	1275 (0.52)	<0.0001^c^		
	C	352 (0.62)	136 (0.41)	1177 (0.48)	0.0073^d^		
	**Genotype**	**(n = 259)**	**(n = 189)**	**(n = 785)**			
**rs16944** *IL1B* Promoter (−511 C/T)	CC	119 (0.46)	115 (0.61)	489 (0.62)	<0.0001^a^ 0.08^b^	0.0001 (AV) 0.07 (SV) 0.1243 (C)	0.4454 (0.3586–0.5532) (AV) 0.9314 (0.7085–1.2244) (SV)
	CT	90 (0.34)	66 (0.35)	270 (0.34)			
	TT	50 (0.20)	8 (0.04)	26 (0.03)			
	Allele						
	C	328 (0.63)	296 (0.78)	1248 (0.79)	<0.0001^c^		
	T	190 (0.37)	82 (0.22)	322 (0.21)	0.06^d^		

‘n’ represents number of Patients/ Controls, HWE refers to Hardy-Weinberg Equilibrium, (AV) refers to Active Vitiligo, (SV) refers to Stable Vitiligo, (C) refers to Controls and CI refers to Confidence Interval.

a Active Vitiligo vs. Controls using the chi-squared test with 3×2 contingency table,

b Stable Vitiligo vs. Controls using the chi-squared test with 3×2 contingency table,

c Active Vitiligo vs. Controls using the chi-squared test with 2×2 contingency table,

d Stable Vitiligo vs. Controls using the chi-squared test with 2×2 contingency table.

Odds ratio is based on allele frequency distribution. Statistical significance was considered at *p* value ≤0.017 due to Bonferroni's correction for multiple testing.

### Linkage disequilibrium and haplotype analysis

The LD analysis revealed that the two polymorphisms investigated in the *NPY* gene were in low LD association (+1128 T/C: −399 T/C; D′ = 0.260, r^2^ = 0.004) (Table 4). Estimated frequencies of haplotypes differed significantly between vitiligo patients and controls (global *p*-value <0.0001). However, the TC haplotype was more frequently observed in vitiligo patients and increased the risk of vitiligo by 2.3-fold [*p*<0.0001; odds ratio (OR): 2.312; 95% confidence interval (CI): (1.790–2.985)] (Table 4).

**Table 4:** Distribution of haplotypes frequencies for *NPY* gene structural and promoter polymorphisms (1128 T/C and −399 T/C) among vitiligo patients and controls.

Haplotype (1128 T/C and −399 T/C)	Vitiligo Patients (Freq. %) (n = 575)	Controls (Freq. %) (n = 1279)	*p* for Association	*p*_(global)_	Odds ratio (95% CI)
CC	1.31 (0.004)	44.94 (0.060)	<0.0001	<0.0001	0.056 (0.010–0.322)
CT	14.69 (0.040)	25.06 (0.034)	0.5690		1.210 (0.627–2.332)
TC	199.69 (0.549)	257.06 (0.345)	<0.0001		2.312 (1.790–2.985)
TT	148.31 (0.407)	418.94 (0.561)	<0.0001		0.537 (0.416–0.692)

‘n’ represents number of Patients/ Controls and CI represents Confidence Interval (frequency <0.03 in both control & case has been dropped and was ignored in analysis).

### Analysis of association between *IL1B* −511 C/T promoter polymorphism and vitiligo

The genotype and allele frequencies for −511 C/T promoter polymorphism of *IL1B* differed significantly between vitiligo patients and controls (*p*<0.0001; *p*<0.0001 respectively). The patient population was deviated whereas the control population was found to be in HWE for this polymorphism (*p* = 0.0002 and *p* = 0.1243 respectively). This study has 90% statistical power for the effect size 0.1 to detect association of the investigated polymorphisms at *p*<0.05. Association studies for *IL1B* promoter (−511; C/T) polymorphism was compared in GV and LV patients from Gujarat (Table 2). The observed genotype and allelic frequencies differed significantly for this polymorphism between GV and LV patients as compared to healthy controls (*p*<0.0001, *p* = 0.0003 and *p*<0.0001, *p* = 0.0218 respectively). Similar studies were performed to compare the AV and LV patients (Table 3). Both genotype and allelic frequencies for *IL1B* promoter polymorphism differed significantly between AV patients and controls (*p*<0.0001 and *p*<0.0001 respectively). However the genotype as well as allelic frequencies for SV patients did not differ as compared to controls (*p* = 0.08 and *p* = 0.06 respectively).

### Relative gene expression of *IL1B* in vitiligo patients and controls

**Figure 1:**
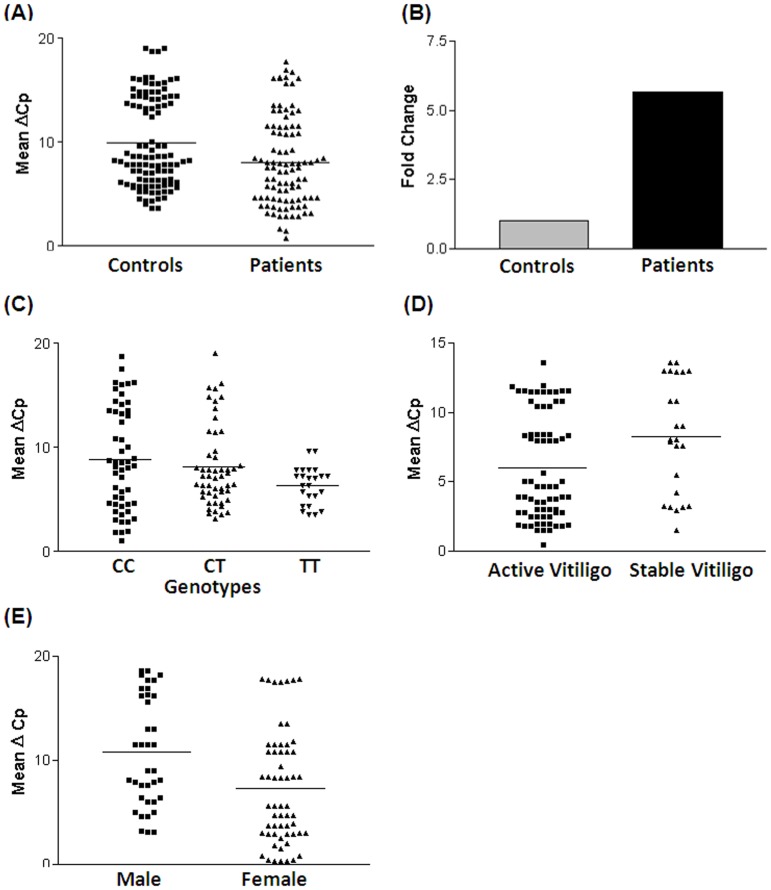
Relative gene expression of *IL1Β* in controls and vitiligo patients. (**A**) Expression of *IL1Β* transcripts in 105 (42 male and 63 female) controls, 95 (37 male and 58 female) vitiligo patients, as suggested by Mean ΔCp. Vitiligo patients showed significantly increased mRNA levels of *IL1Β* as compared to controls (Mean ΔCp±SEM: 9.730±0.5521 vs 7.187±0.6169; *p* = 0.003). (**B**) Expression fold change of *IL1Β* transcripts in 95 vitiligo patients against controls 105 showed 5.83 fold higher expression as determined by 2^−ΔΔCp^ method. (**C**) Expression of *IL1Β* transcripts with respect to *IL1Β* C/T (rs16944) promoter polymorphism as suggested by Mean ΔCp. Individuals with TT genotype showed significantly increased mRNA levels of *IL1Β* as compared to individuals having CC genotype (Mean ΔCp±SEM: 6.358±0.3659 vs 8.829±0.6770; *p* = 0.019) and CT genotype (Mean ΔCp±SEM: 6.358±0.3659 vs 8.136±0.5390; *p* = 0.036). There was no significant difference in the expression of *IL1Β* between individuals with CC genotype as compared to individuals with CT genotype (Mean ΔCp±SEM: 8.829±0.6770 vs 8.136±0.5390; *p* = 0.426). Cochran-Armitage trend analysis revealed significant difference between patients and controls (Odds ratio  = 0.574, Chi-square  = 28.67, *p* = 8.588e-^08^) for *IL1B* transcript levels with respect to different genotype categories. ANOVA's trend test confirmed a significant decrease in the mean ΔCp values for patients (*p* = 0.035), as compared to controls (*p* = 0.154). (**D**) Expression of *IL1Β* transcripts with respect to activity of the disease in 71 (28 male and 43 female) patients with active vitiligo and 24 (10 male and 14 female) patients with stable vitiligo, as suggested by Mean ΔCp. Active vitiligo patients showed significantly increased mRNA levels of *IL1Β* as compared to stable vitiligo patients (Mean ΔCp±SEM: 4.720±0.7307 vs 8.285±1.289; *p* = 0.015). (**E**) Expression of *IL1Β* transcripts with respect to gender differences in 37 male patients and 58 female patients with vitiligo, as suggested by Mean ΔCp. Female patients with vitiligo showed significantly increased mRNA levels of *IL1Β* as compared to male vitiligo patients (Mean ΔCp±SEM: 10.84±1.203 vs 6.808±1.255; *p* = 0.026).

The *IL1B* transcript levels were compared in 95 vitiligo patients and 105 age matched unaffected controls after normalization with *GAPDH* transcript levels. The *IL1B* transcript levels in vitiligo patients were significantly higher than in controls (*p* = 0.003) as suggested by mean ΔCp values (Fig. 1A). The 2^−ΔΔCp^ analysis showed approximately 6 fold higher expression of *IL1B* transcript in patients as compared to controls (Fig. 1B).

### Correlation of *IL1B* transcript levels with −511 C/T promoter polymorphism

Expression of *IL1Β* with respect to *IL1Β* C/T (rs16944) promoter polymorphism revealed that individuals with TT genotype showed significantly increased *IL1Β* mRNA levels as compared to individuals having CC (*p* = 0.019) and CT genotypes (*p* = 0.036). There was no significant difference in the expression of *IL1Β* between individuals with CC genotype as compared to individuals with CT genotype (*p* = 0.426) (Fig. 1C). Further, we performed Cochran-Armitage trend test for analyzing the trend of *IL1B* transcript levels with respect to −511 C/T promoter polymorphism genotypes for each group individually. We found significant difference in *IL1B* transcript levels between vitiligo patients and controls (Odds ratio = 0.574, Chi-square = 28.67, *p* = 8.588e-^08^) with respect to different genotypes. Moreover, ANOVA's trend test was used to see the change in mean ΔCp values across the different −511 C/T promoter genotypes. The analysis revealed a significant decrease in the mean ΔCp values for patients (*p* = 0.035), as compared to controls (*p* = 0.154).

### Effect of *IL1B* expression on disease progression

The transcript levels of *IL1B* were analyzed with respect to progression of disease in 71 patients with active vitiligo and 24 patients with stable vitiligo. As suggested by mean ΔCp, AV patients showed significant increase in *IL1B* mRNA levels as compared to SV patients (*p* = 0.015, Fig. 1D). The elevated levels of *IL1B* transcript in patients with AV suggest that *IL1B* may be involved in progression of the disease.

### Gender based analysis of *IL1B* expression

Expression of *IL1B* transcripts was analyzed with respect to gender differences in 37 male patients and 58 female patients with vitiligo. The female patients showed significant increase in *IL1B* mRNA levels as compared to male vitiligo patients (*p* = 0.026, Fig. 1E). However, we did not find any significant difference in *IL1B* mRNA levels between AV and SV patients based on gender distribution (data not shown).

## Discussion

Melanocytes are neural crest derived cells with an embryological link to the nervous system [21]. Neurochemical hypothesis of vitiligo pathogenesis is based on certain abnormalities observed in depigmented skin such as perturbed acetylcholine esterase activity, neuropeptide distribution and catecholamine metabolism [22]. Previously, we reported decrease in acetylcholine esterase activity in vitiligo patients as compared to controls suggesting the involvement of neural factors in pathogenesis of vitiligo [23].

In the present study, we have investigated structural and promoter polymorphisms of *NPY* which are reported to have strong association with higher levels of NPY. We found that Leu7Pro (exon 2 +1128 T/C) polymorphism as well as (−399 T/C) promoter polymorphism of *NPY* were significantly associated with vitiligo susceptibility. Moreover, the observed genotype and allele frequencies for these polymorphisms in GV and LV patients were found to be significantly different as compared to controls (Table 2, Table 3). The polymorphisms were also found to be in association with active and stable vitiligo as compared to controls (Table 3). Exon 2 +1128 T/C (Leu7Pro) SNP is located in the signal peptide region which influences the intracellular processing of preproNPY peptide, resulting in altered processing of the prohormone and storage or kinetics of NPY release [24]. The Leu7Pro polymorphism has shown to confer susceptibility to development of diabetic nephropathy in Swedish female type 1 diabetes patients [25]. Moreover, individuals with Leu7/Pro7 genotype have an average 42% maximal increase in plasma NPY as compared to Leu7/Leu7 individuals [24]. The promoter (−399 T/C) polymorphism of *NPY* was suspected to be responsible for inter-individual variation in resiliency to stress, a risk factor for many diseases [26]. Moreover, elevated serum levels of NPY were detected in patients with asthma and systemic lupus erythematosus suggesting its role in acute inflammatory diseases [27]–[28]. Reactivity against NPY antibody was found to be higher in vitiligo patients in lesional and marginal areas during immunoreactivity studies with a neuronal marker suggesting that NPY may be involved in vitiligo pathogenesis [29]. Caixia *et al.* [30] reported increase in NPY levels from skin lesion and plasma of vitiligo patients compared to unaffected controls, suggesting the involvement of NPY in vitiligo pathogenesis.

The catecholamine secretion by chromaffin cells is regulated by neuropeptides and cytokines that are co-released with catecholamines or are present in circulating blood [31]–[33]. Further experimental evidences suggested that the role of IL1B is not limited to catecholamine release from chromaffin cells, but is also involved in neuropeptide biosynthesis [34]–[35]. Joana *et al.* [9] also showed that IL1B plays a critical role in biosynthesis and release of NPY which in turn induces the catecholamine release. L-tyrosine serves as a precursor to melanin pigment, catecholamines and thyroid hormones [36] and L-dihydroxyphenylalanine (L-DOPA) serve as substrates and intermediates of melanogenesis [37]. Depending on genetic makeup of the individual and environmental factors, L-tyrosine and L-DOPA can act as inducers or modifiers of melanogenesis and melanocytic phenotype [38]. It has been suggested that nitrification of L-tyrosine, and other byproducts of melanogenesis, may serve both as a protective means for the cell survival and as an inducer of apoptosis [38]. Moreover, L-DOPA stimulates nitric oxide synthetase [39], and nitrate levels which can serve as markers of nitrosative stress in vitiligo [40]. Such mechanism might play a role in neurodegenerative diseases including vitiligo [39], [41], [42]. In addition, L-tyrosine and L-DOPA both are positive regulators of melanocyte-stimulating hormone (MSH) receptor activity [43], [44]. A dual hormonal control of color change is regulated by MSH and melanin-concentrating hormone (MCH) [45]. Melanin-concentrating hormone receptor 1 (MCHR1) expression has been detected in human melanocytes. Importantly, MCHR1 has been identified as an autoantigen in vitiligo patients [46]. It has been shown that MCH stimulation in cultured human melanocytes, reduced α-MSH-induced increase in cAMP production. Moreover, the melanogenic actions of α-MSH were inhibited by MCH [47]. Therefore, the MCH/MCHR1 system may regulate skin pigmentation through modifications of melanocortin signaling.

It is worth noting that in addition to neuropeptide Y, the neuroendocrine modulators controlling the activity of the hypothalamus-pituitary-adrenal axis are expressed in the skin including CRH, urocortin, and POMC, with its products ACTH, alpha-MSH, and beta-endorphin. These neuroendocrine modulators are produced in the skin under stress [48]–[50]. Therefore, it has been suggested that skin neuroendocrine system communicates with itself and with the systemic level through humoral and neural pathways to induce vascular, immune, or pigmentary changes, to directly buffer noxious agents or neutralize the elicited local reactions [48]. Recently, Slominski *et al*. [50] has suggested that expression of skin cells producing proinflammatory CRH and anti-inflammatory POMC derived peptides along with CRFR-1α is environmentally regulated, and their dysfunction can lead to skin and systemic diseases that worsen with stress. Environmentally stressed skin can activate both the central and local HPA axis through either sensory nerves or humoral factors to turn on homeostatic responses counteracting cutaneous and systemic environmental damage [50], [51].

There are reports that IL1B evokes catecholamine synthesis by activating the enzyme tyrosine hydroxylase (TH) by Ser40 phosphorylation through different intracellular pathways such as protein kinase C (PKC), protein kinase A (PKA), and mitogen-activated protein kinase (MAPK) [23], [52], [53]. Increased catecholamines inhibits mitochondrial calcium uptake which results in generation of free radicals [54]. Moreover, IL1B is a potent activator of superoxide dismutase 2 (SOD2) in different tissues and cell types [55]; which could lead to increased H_2_O_2_ production as observed in vitiligo patients. Interestingly, our recent study suggests increased levels of *SOD2* transcripts and SOD2 activity in vitiligo patients which might be one of the consequences of increased IL1B [56].

In the present study, we show that −511C/T promoter polymorphism of *IL1B* gene is associated with vitiligo in Gujarat population (Table 1). Moreover, higher frequency of TT and CT genotypes in patients than that of controls indicating the profound effect of allele ‘T’ with susceptibility to vitiligo. The observed genotype and allelic frequencies differed significantly for this polymorphism between generalized and localized vitiligo patients with respect to healthy controls. The genotype and allele frequencies differed significantly in active and stable vitiligo patients, suggesting association of this polymorphism with the disease progression (Table 3). Interestingly, the mRNA levels of *IL1B* in patients were found to be significantly higher than controls suggesting the possible involvement of IL1B in vitiligo pathogenesis (Fig. 1A). The −511 T allele is reported to increase the *IL1B* mRNA levels [57], [58]. Further, genotype-phenotype correlation analysis revealed that individuals with TT genotype showed significantly increased mRNA levels of *IL1Β* as compared to individuals having CC genotype (*p* = 0.019) and CT genotype (*p* = 0.036), which shows disease-independent effect of rs16944 on *IL1B* mRNA level. However, there was no significant difference in the expression of *IL1Β* between individuals with CC genotype as compared to individuals with CT genotype (*p* = 0.426) (Fig. 1C). Furthermore, Cochran-Armitage trend analysis demonstrated significant increase in *IL1B* transcript levels in vitiligo patients with respect to the different genotypes compared to those of controls (*p* = 8.588^e-08^). Moreover, ANOVA's trend test confirmed and revealed a significant decrease in the mean ΔCp values for patients (*p* = 0.035), as compared to controls (*p* = 0.154). Our results showed that the genotype and allele frequencies for -511C/T promoter polymorphism of *IL1B* differed significantly in active and stable vitiligo patients (Table 3), suggesting association of this polymorphism with the disease progression. Moreover frequency of the susceptible allele −511 T is significantly lesser in stable cases as compared to active cases of the disease which might be one of the reasons to have effect of this SNP with the active form of the disease. In addition, our results also demonstrated increased *IL1B* mRNA levels in active vitiligo as compared to stable cases (Fig. 1D), further confirming the important role of IL1B in disease progression. Higher *IL1B* mRNA levels are seen in female patients compared to male patients (Fig. 1E). However, we did not find any significant difference between active and stable vitiligo patients based on gender distribution (data not shown).

Our previous studies suggest that susceptibility to generalized vitiligo involves a number of immune regulatory genes such as cytotoxic T-lymphocyte associated antigen-4 (*CTLA*4), human leukocyte antigen (*HLA*), interleukin-4 (*IL*4), tumor necrosis factor-α (*TNFA*), tumor necrosis factor-β (*TNFB*), interferon-gamma (*IFNG*), melanocyte proliferating gene 1 (*MYG1*), and NACHT leucine-rich-repeat protein 1 (*NLRP1*) [59]–[67]. For the first time we report an association between *IL1B* promoter polymorphism and vitiligo along with higher transcript levels of *IL1B* in vitiligo patients as compared to controls. NPY synthesis and release is also governed by IL1B [9]. Taken together, altered IL1B transcript levels due to genetic variability in IL1B might be associated with elevated NPY levels in patients with vitiligo. Furthermore, Joana *et al.* [9] suggested that IL1B induces biosynthesis and release of NPY which in turn induces chromaffin cells to secrete catecholamines which enter the blood flow and interacts with α-adrenergic receptors resulting in vasoconstriction. Repeated vasoconstriction of blood vessels leads to epidermal and dermal hypoxia with hyper production of toxic oxyradicals by different pathways which might lead to the destruction of melanocytes [5]. Recent animal studies, *in-vitro* cultures and clinical trials provide evidence that a causative role for IL1B as the primary agonist in the loss of beta-cell mass in type 2 diabetes. *In vitro*, IL1B mediated autoinflammatory process results in beta-cell death [27].

Wheway *et al.* [10] provided new insights into the role of NPY in innate immunity by showing NPY autocrine stimulation pathway on macrophages. NPY signaling on dendritic cells promotes increased antigen uptake and IL12 secretion while on T cells it promotes Th2 responses by increasing IL4 production. Hernanz *et al.* [68] reported that NPY enhanced the production of IL6 and TNFα in peripheral blood cells. In addition, our previous studies have shown high levels of serum IL4 and TNFα as well as increased mRNA levels in patients with vitiligo compared to controls [62], [63] suggesting that NPY might be involved in the cell-mediated as well as humoral immune mechanisms and plays a crucial role in melanocyte destruction. However, the exact role of NPY in vitiligo pathogenesis is yet to be elucidated. Similarly, higher IL1B levels can cause autoimmune responses against melanocytes, by initiating a cascade of reactions involving inflammosome formation and ultimately resulting in vitiligo. NLRP1 is a key regulator of the innate immune system, particularly in the skin and the NLRP1 inflammasome promotes caspase 1 dependent cleavage of IL1B precursor in to bioactive IL1B, resulting in IL1B secretion and downstream inflammatory responses. Interestingly, our recent study shows significant association of *NLRP1* promoter variants with GV and *NLRP1* overexpression in patients compared to controls; which might lead to increased IL1B production in vitiligo patients [67].

IL1B can act on lymphocytes by up regulating IL2 receptor expression, prolonging survival of T cells, enhancing antibody production by B cells and increasing B cell proliferation [69], [70]. IL1B also plays a crucial role in driving the differentiation of Th17 and Th1 cells, ultimately evoking immune response against melanocytes [71]. Our recent studies have shown decrease in regulatory T-cells, increase in CD8^+^ T-cells and anti-melanocyte antibody levels in GV patients which were positively correlated with disease onset and progression [72], [73].

In conclusion, our findings suggest that *NPY* exon 2 +1128 T/C, −399 T/C and *IL1B* −511 C/T promoter polymorphisms are significantly associated with vitiligo susceptibility, which might result in higher levels of IL1B thereby leading to autoimmune mediated responses in vitiligo. In addition, the study also emphasizes the influence of *NPY* and *IL1B* genes on disease progression for developing vitiligo.

## Supporting Information

Figure S1 (**A**) PCR-RFLP analysis of *NPY* (rs16139 T/C) exon 2 polymorphism on 2.5% agarose gel electrophoresis: lanes: 1, 3 & 4 show heterozygous (TC) genotypes; lanes: 2, 5 & 6 show homozygous (TT) genotypes; lane: 7 shows 100 bp DNA ladder. (**B**) PCR-RFLP analysis of *NPY* (rs16147 T/C) promoter polymorphism on 10% polyacrylamide gel electrophoresis: lanes: 1 & 5 show homozygous (TT) genotypes; lanes: 2 & 6 show heterozygous (TC) genotypes; lane: 3 shows homozygous (CC) genotype; lane 4 shows 100 bp DNA ladder. (**C**) PCR-RFLP analysis of *IL1Β* (rs16944) promoter polymorphism on 2.5% agarose electrophoresis: lanes: 1 & 6 show homozygous (CC) genotypes; lanes: 2, 3 & 5 show heterozygous (CT) genotypes; lane: 4 shows homozygous (TT) genotype.
(TIF)

Table S1 Demographic characteristics of vitiligo patients and unaffected controls.
(DOC)

Table S2 Primers and restriction enzymes used for genotyping of *NPY, IL1B* SNPs and *IL1B* gene expression.
(DOC)
