# Supplementary figures and images for: Association of Neuropeptide Y (NPY), Interleukin-1B (IL1B) Genetic Variants and Correlation of IL1B Transcript Levels with Vitiligo Susceptibility
Source: PLoS One. 2014 Sep 15;9(9):e107020. doi: 10.1371/journal.pone.0107020 (PMC4164539; doi:10.1371/journal.pone.0107020)

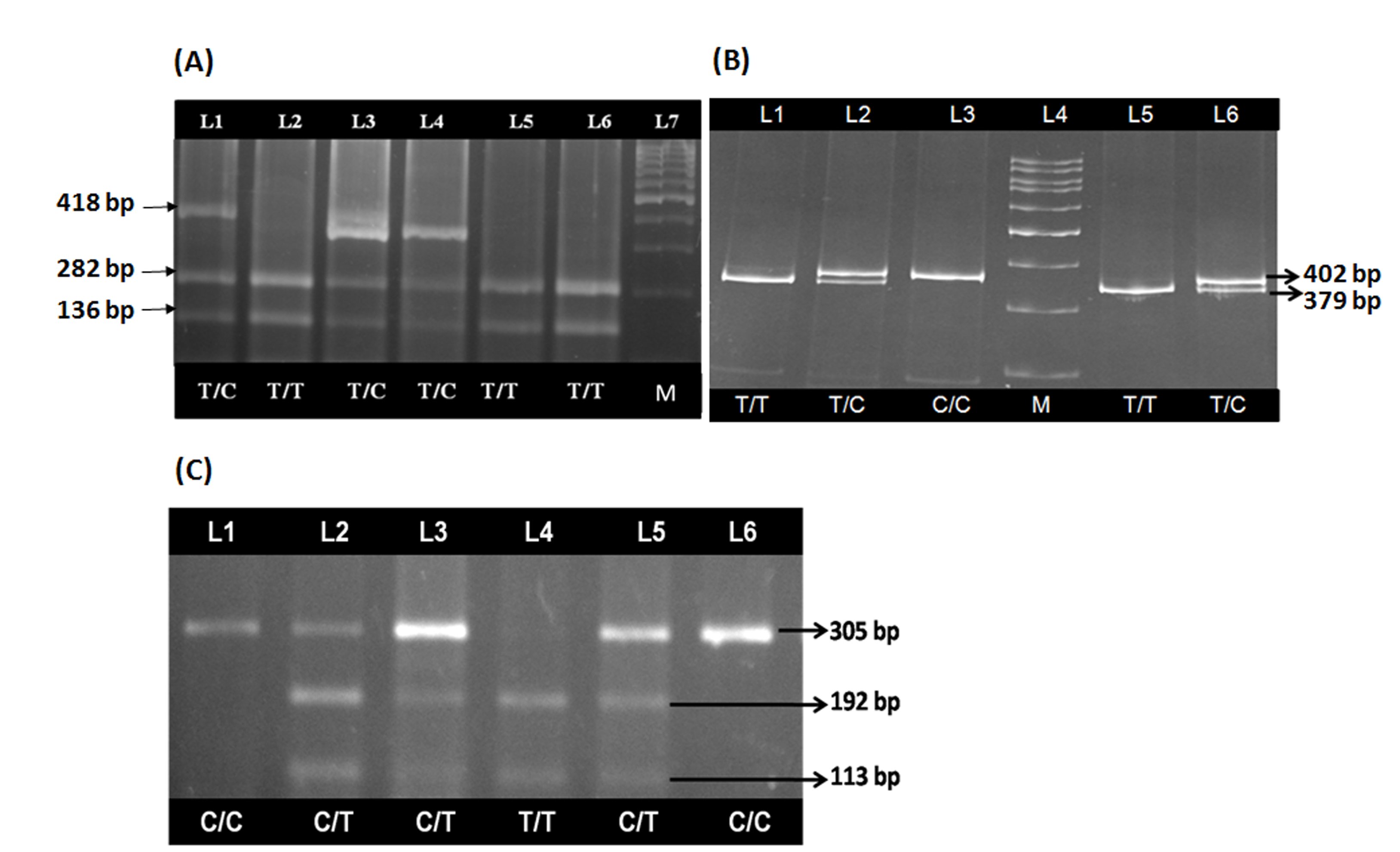

Supplement: Figure S1 — (A) PCR-RFLP analysis of NPY (rs16139 T/C) exon 2 polymorphism on 2.5% agarose gel electrophoresis: lanes: 1, 3 & 4 show heterozygous (TC) genotypes; lanes: 2, 5 & 6 show homozygous (TT) genotypes; lane: 7 shows 100 bp DNA ladder. (B) PCR-RFLP analysis of NPY (rs16147 T/C) promoter polymorphism on 10% polyacrylamide gel electrophoresis: lanes: 1 & 5 show homozygous (TT) genotypes; lanes: 2 & 6 show heterozygous (TC) genotypes; lane: 3 shows homozygous (CC) genotype; lane 4 shows 100 bp DNA ladder. (C) PCR-RFLP analysis of IL1Β (rs16944) promoter polymorphism on 2.5% agarose electrophoresis: lanes: 1 & 6 show homozygous (CC) genotypes; lanes: 2, 3 & 5 show heterozygous (CT) genotypes; lane: 4 shows homozygous (TT) genotype. (TIF) [file pone.0107020.s001.tif]
