# Supplementary material for: Association of Neuropeptide Y (NPY), Interleukin-1B (IL1B) Genetic Variants and Correlation of IL1B Transcript Levels with Vitiligo Susceptibility
Source: PLoS One. 2014 Sep 15;9(9):e107020. doi: 10.1371/journal.pone.0107020 (PMC4164539; doi:10.1371/journal.pone.0107020)
Supplement: Table S1 — Demographic characteristics of vitiligo patients and unaffected controls. (DOC) [file pone.0107020.s002.doc]

**Table S1.** Demographic characteristics of vitiligo patients and unaffected controls.

|  | **Vitiligo Patients** |  | **Controls** |
| --- | --- | --- | --- |
| Average age  (mean age ± SD)  Sex: male  female  Onset age  (mean age ± SD)  Duration of disease  (mean ± SD)  Type of disease:  Generalized  Localized  Family history  Active  Stable | (n = 575) |  | (n = 1279) |
|  | 31.95 ± 12.53 yrs  256 (44.51%)  319 (55.49%)  22.13 ± 11.85 yrs  8.1 ± 6.7 yrs  335 (58.26%)  240 (41.74%)  45 (13.12%)  430 (74.78%)  145 (25.22%) |  | 29.32 ± 13.24 yrs  552 (43.16%)  727 (56.84%)  NA  NA  NA    NA  NA  NA |
