# Supplementary material for: Association of Neuropeptide Y (NPY), Interleukin-1B (IL1B) Genetic Variants and Correlation of IL1B Transcript Levels with Vitiligo Susceptibility
Source: PLoS One. 2014 Sep 15;9(9):e107020. doi: 10.1371/journal.pone.0107020 (PMC4164539; doi:10.1371/journal.pone.0107020)
Supplement: Table S2 — Primers and restriction enzymes used for genotyping of NPY, IL1B SNPs and IL1B gene expression. (DOC) [file pone.0107020.s003.doc]

**Table S2.** Primers and restriction enzymes used for *NPY , IL1B* SNPs genotyping and *IL1B*  gene expression.

| **Gene/SNP Primer** | **Sequence** | **Annealing**  **Temperature** | **Amplicon Restriction**  **size Enzyme** |
| --- | --- | --- | --- |
| **(rs16139)**  *NPY* +1128 T/C F  *NPY* +1128 T/C R    **(rs16147)**  *NPY* -399 T/C F  *NPY* -399 T/C R  **(rs16944)**  *IL1B* -511 C/T F  *IL1B* -511 C/T R  *IL1B*  Expression F  *IL1B* Expression R  *GAPDH* Expression F  *GAPDH* Expression R | 5’- ATTGGGGGTCGCGTGTGGTAG -3’  5’- GTCCTGCCCTGGGATAGAGCG -3’  5’- TTCCTACTCCGGCACCCAGTGAG -3’  5’- GGGCTTTTATGGAGCTTCCTCGC -3’  5’- GTTTAGGAATCTTCCCACTT-3’  5’- TGGCATTGATCTGGTTCATC-3’  5’-AGATGAAGTGCTCCTTCCAGG-3’  5’-TGGTCGGAGATTCGTAGCTG-3’  5’-ATCCCATCACCATCTTCCAGGA-3’  5’- CAAATGAGCCCCAGCCTTCT-3’ | 60°C  63°C  63°C  65°C  65°C | 402 bp *BseN*I    417 bp  *Alu*I    305 bp *Bsu36I*  153 bp -  122 bp - |
|  |  |  |
